# Identification of a native *Bacillus thuringiensis* strain from Sri Lanka active against Dipel-resistant *Plutella xylostella*

**DOI:** 10.7717/peerj.7535

**Published:** 2019-08-23

**Authors:** Rashini Yasara Baragamaarachchi, Jayanetti Koralage Ramani Radhika Samarasekera, Ovitigala Vithanage Don Sisira Jagathpriya Weerasena, Kurt Lamour, Juan Luis Jurat-Fuentes

**Affiliations:** 1 Institute of Biochemistry, Molecular Biology and Biotechnology, University of Colombo, Colombo, Sri Lanka; 2 Industrial Technology Institute, Colombo, Sri Lanka; 3 Department of Entomology and Plant Pathology, University of Tennessee, Knoxville, TN, United States of America

**Keywords:** *Bacillus thuringiensis*, *Plutella xylostella*, Cry toxin, Resistance

## Abstract

**Background:**

Biopesticides based on strains of the bacterium *Bacillus thuringiensis* (Bt) are used globally for effective and environmentally friendly pest control. The most serious threat to the sustainable use of these microbial pesticides is the development of resistance on targeted pests. Populations of *Plutella xylostella* (diamondback moth) have evolved field resistance to Bt pesticides at diverse locations worldwide. Discovery of novel Bt strains with varied toxin profiles that overcome resistance is one of the strategies to increase sustainability of Bt pesticides against *P. xylostella*. In this study, we report isolation and characterization of a Bt strain named AB1 from Sri Lanka displaying toxicity towards larvae of *P. xylostella* resistant to the commercial Bt pesticide Dipel.

**Methods:**

Strains of Bt from diverse environments in Sri Lanka were evaluated for protein crystal production through Differential Interference Contrast (DIC) microscopic examination, and for insecticidal activity against *P. xylostella* in bioassays. The genome of the AB1 strain was sequenced by Hiseq Illumina sequencing to identify the insecticidal genes present in the genome and nano liquid chromatography followed by tandem mass spectrometry (nanoLC/MS/MS) of purified crystal proteins of AB1 was performed to identify the expressed insecticidal proteins. Multilocus sequence typing and *Gyrase B* gene sequence analyses were performed to identify the phylogenetic origin of the AB1 strain.

**Results:**

The AB1 strain was identified as producing high levels of bipyramidal crystals and displaying insecticidal activity against susceptible and Dipel-resistant strains of *P. xylostella*. Multilocus sequence typing and phylogenetic analysis of the *Gyrase B* gene identified that AB1 belongs to the *B. thuringiensis* subsp. *aizawai* serotype*.* Comparative analysis of genomic and proteomic data showed that among the insecticidal protein coding genes annotated from the AB1 genome (*cry*1Aa, *cry*1Ca, *cry*1Da, *cry*1Ia, *cry*2Ab and* cry*9), Cry1Ca and Cry1Da toxins represented most of the toxin fraction in parasporal crystals from AB1. Overall findings warrant further development of *B. thuringiensis* subsp. *aizawai* AB1 strain as a pesticide to control *P. xylostella*.

## Introduction

Strains of the bacterium *Bacillus thuringiensis* (Bt) produce a number of parasporal crystal proteins with insecticidal activity. Their high specificity and associated environmental safety have contributed to make Bt the most successfully commercialized biocontrol agent worldwide (Glare et al., 2012; Jurat-Fuentes & Crickmore, 2017). For example, the Dipel pesticide is based on *B. thuringiensis* subsp. *kurstaki* HD-1, which produces insecticidal proteins Cry1Aa, Cry1Ab, Cry1Ac, Cry2Aa and Cry2Ab as active ingredients (Höfte & Whiteley, 1989; Abbott Laboratories, 1992) to control lepidopteran larvae. The mode of action of these Cry toxins includes a number of successive steps, yet binding of the toxin to receptors on the midgut epithelium in the host is considered the most critical given that high levels of resistance are commonly linked to reduced toxin binding (Jurat-Fuentes & Crickmore, 2017). This resistance represents the main threat to the sustainability of Bt pesticides.

*Plutella xylostella* (Diamondback moth, Lepidoptera: Plutellidae) is a destructive agricultural pest attacking crucifer crops worldwide (Talekar & Shelton, 1993; Furlong, Wright & Dosdall, 2013) that is controlled by Bt pesticides. Annual global crucifer yield loss and control costs of *P. xylostella* together represent USD 2.7 billion (Zalucki et al., 2012; Furlong, Wright & Dosdall, 2013). Its high reproductive potential, voracious feeding, high genetic elasticity, and cosmopolitan distribution makes *P. xylostella* difficult to control (Machekano, Mvumi & Nyamukondiwa, 2017). Moreover, populations of *P. xylostella* have developed field-evolved resistance to virtually all used insecticides, including Bt pesticides such as Dipel (Kirsch & Schmutterer, 1988; Tabashnik et al., 1996; Heckel et al., 2001; Zhao et al., 2002; Furlong, Wright & Dosdall, 2013). Since resistance to Dipel was associated with reduced binding of Cry1A toxins (Ferre et al., 1991) it is expected that the use of *B. thuringiensis* strains or transgenic crops producing insecticidal proteins binding to distinct sites from those of Cry1A toxins would be effective against Dipel-resistant *P. xylostella* and assist in delaying resistance evolution (Zhao et al., 2003).

In this study, we report the identification of a *B. thuringiensis* strain from Sri Lankan soil displaying insecticidal activity against *P. xylostella*, including a Dipel-resistant strain (NO-QAGE). The sequenced genome of this strain was used as a reference database for proteomic identification of insecticidal proteins present in its parasporal body. The results support that toxicity of this strain against the NO-QAGE strain of *P. xylostella* is related to the production of Cry toxins not sharing binding sites with Cry1A toxins produced by *B. thuringiensis* subsp. *kurstaki.*

## Materials & Methods

### Bacterial strains and Plasmids

Reference strains *B. thuringiensis* subsp. *kurstaki* HD-1 (ATCC 33679) and *B. thuringiensis* subsp. *israelensis* (ATCC 35646) were purchased from the American Type Culture Collection (ATCC, Manassas, VA, USA). Strains, *B. thuringiensis* subsp. *aizawai* HD-133 (BGSC 4J1, 4J3) and *B. thuringiensis* subsp. *tenebrionis* (BGSC 4AA1) were purchased from the *Bacillus* Genetic Stock Center (BGSC, Ohio State University, Columbus, OH, USA). The TA cloning vector pGEM-T Easy used in this study was purchased from Promega (Madison, WI, USA).

### Isolation and identification of *Bacillus thuringiensis* strains

Previously, *B. thuringiensis* strains were isolated from 63 soil samples from natural habitats in Sri Lanka (Travers, Martin & Reichelderfer, 1987). Isolates were designated with given numbers and are stored at the Industrial Technology Institute *B. thuringiensis* collection (Colombo, Sri Lanka). Isolated spore-forming *Bacilli* were grown on chromogenic *Bacillus* agar (HiMedia, Mumbai, India), to identify putative Bt colonies based on their blue–green color and irregular margins.

Putative Bt isolates were sub-cultured on Luria-Bertani (LB) agar plates and incubated at 30 ± 2 °C for 16 h to obtain pure cultures, which were used for genomic DNA extraction using the PureLink Genomic DNA Mini Kit (Invitrogen), following manufacturer’s instructions. The gyrase B (*gyrB*) gene was amplified from genomic DNA using nested PCR primers gyrB-F1 (5′ATGGAACAAAAGCAAATGCA-3′) and gyrB-R1 (5′TTAAATATCAAGGTTTTTCA-3′), and gyrB-F2 (5′-CCTTGYTTTGCWGAWCCDCC-3′) and gyrB-R2 (5′ACWCGTATGCGTGARYTRGC-3′) as second primer pair (Soufiane & Côté, 2009). The first PCR was carried out in 25 µL containing template genomic DNA (250 ng), 1× reaction buffer, 200 µM dNTPs, 1 mM MgCl_2_, 0.8 µM (each) primer and 1.25 U of *Taq* DNA polymerase (Promega). Amplification included initial denaturation at 95 °C for 3 min followed by 30 amplification cycles of denaturation at 95 °C for 30 s, annealing at 45 °C for 30 s and extension at 72 °C for 30 s in a Veriti thermocycler (Applied Biosystems, USA). A final extension step of 7 min at 72 °C was added after completion of 30 cycles. The second PCR was performed in a 50 µL reaction including 2 µL of the amplicon from the first PCR as template DNA, 1× reaction buffer, 200 µM dNTPs, 1 mM MgCl_2,_ 0.8 µM (each) primer and 2.5 U of *Taq* DNA polymerase (Promega). Amplification included initial denaturation at 95 °C for 3 min followed by 30 amplification cycles of denaturation at 95 °C for 45 s, annealing at 50 °C for 30 s and extension at 72 °C for 1 min. A final extension step of 7 min at 72 °C was added after completion of 30 cycles. Amplified products were purified from 1% low-melting agarose using PureLink Quick Gel Extraction Kit (Invitrogen, Waltham, MA, USA) according to the manufacturer’s instructions. Purified amplicons of *gyrB* were bi-directionally sequenced using the Big Dye Terminator Cycle sequencing kit in an Applied Biosystems 3500 dx genetic analyzer (Applied Biosystems). Forward and reverse sequences were aligned and edited using the BioEdit sequence alignment editor (version 7.2.5). The sequences were used as query over the GenBank database at NCBI using BLAST searches.

### Detection of insecticidal protein genes

Polymerase chain reaction (PCR) with specific primers was used to detect insecticidal genes (c*ry*, *vip* and *cyt*) in isolated Bt strains. Strains were cultured in 10 mL of LB broth enriched with salts (0.2% NH_4_SO_4_, 1.4% K_2_HPO_4_, 0.6% KH_2_PO_4_, 0.1% sodium citrate, 0.02% MgSO_4_.7H_2_O) and 0.5% glucose at 30 °C, shaking at 200 rpm for 17 h, and then plasmid DNA was extracted (Reyes-Ramırez & Ibarra, 2008). Reference strains; *B. thuringiensis* subsp. *kurstaki* HD-1 (ATCC 33679), *B. thuringiensis* subsp. *israelensis* (ATCC 35646), *B. thuringiensis* subsp. *aizawai* HD-133 (BGSC 4J1, 4J3) and *B. thuringiensis* subsp. *tenebrionis* (BGSC 4AA1) served as controls in PCR reactions. *Bacillus thuringiensis* strains were screened for lepidopteran-specific *cry* and *vip* genes (*cry*1, *cry*2, *cry*9 and *vip*3A), Coleopteran-specific *cry* genes (*cry*3, *cry*7 and *cry*8), and Dipteran-specific *cry* and *cyt* genes (*cry*4, *cry*10, *cry*11, *cyt*1 and *cyt*2), using universal and gene specific primers (Ben-Dov et al., 1997; Ben-Dov et al., 1999; Bravo et al., 1998; Ejiofor & Johnson, 2002; Porcar & Juárez-Pérez, 2003). Each amplification reaction (25 µL) contained 200 µM dNTP, 0.5 µM of each primer, 2.5 mM MgCl_2_, and 2.5 U of Taq DNA polymerase. Amplifications were carried out in thermocycler following a program of initial denaturation at 94 °C for 3 min, followed by 35-cycles of denaturation at 94 °C for 45 s, annealing at corresponding annealing temperatures of the primer (Table S1) for 45 s and an extension at 72 °C for 1 min. A final extension step of 7 min at 72 °C was included after completion of 35 cycles (Ben-Dov et al., 1997; Bravo et al., 1998). Reaction products were resolved in 1% agarose gels and examined for the presence of the expected amplicon size.

### Observation and purification of insecticidal crystal proteins (ICPs)

*Bacillus thuringiensis* strains were grown on 1/3 Tryptic Soy Broth (TSB) agar for 3 days at 28 °C until sporulation. Individual colonies were suspended in a drop of sterile distilled water and observed in a Differential Interference Contrast (DIC) microscope to document the presence of crystals.

For production of ICPs from *B. thuringiensis* strain AB1, three colonies of a sporulated culture were inoculated separately into 1 mL of sterile distilled H_2_O, vortexed to homogenize and heated at 70 °C for 45 min to kill vegetative cells and synchronize the culture. This pre-inoculum (500 µL) was used to inoculate 500 mL of 1/3 TSB, which were then incubated at 28 °C with shaking at 160 rpm for 3 days until 90% of cells appeared sporulated under microscopic inspection. Parasporal crystal proteins were isolated from spores using solubilization in 50 mM Na_2_CO_3_ containing 0.1% 2-mercaptoethanol, as described elsewhere (Perera et al., 2009). Solubilized crystal proteins were dialyzed overnight against 2 L Na_2_CO_3_ buffer (50 mM) with two changes of buffer, to eliminate 2-mercaptoethanol, and then stored at 4 °C until used (within 1 week).

Proteins present in the isolated parasporal crystal protein mixtures from strain AB1 were analyzed using electrophoresis. Aliquots (10 µL) of each protein sample were mixed with equal volume of 2% electrophoresis buffer (100 mM Tris pH 6.8, 4% SDS, 20% glycerol, 0.2% bromophenol blue, 2% *β*-mercaptoethanol), and heat-denatured for 10 min before loading on lanes of an SDS-10% PAGE gel. After the electrophoresis run, proteins were detected by staining with ProtoBlue Coomassie stain (National Diagnostics, Atlanta, GA, USA).

### Insects and bioassays

Eggs of Dipel susceptible and resistant (NO-QAGE) strains of *P. xylostella* were purchased from Benzon Research (Carlisle, PA). The NO-QAGE strain was derived from a strain with field-evolved resistance to Dipel from Hawaii (Tabashnik et al., 1990), and it displays 500-1,000-fold resistance to *B. thuringiensis* subsp. *kurstaki* (https://www.benzonresearch.com/species-detail). Eggs were placed in an incubator (27 °C, 21% relative humidity, and 16-h light/8-h dark photoperiod) until hatched.

Insecticidal activity of isolated solubilized crystal proteins was evaluated first against Dipel-susceptible *P. xylostella* larvae using surface-diet contamination bioassays. Freshly prepared meridic diet (General Lepidopteran Diet; Frontier Agricultural Sciences, Newark, DE, USA) was dispensed into 128 well bioassay trays (CD International, Pitman, NJ) and allowed to solidify. Toxin (crystal protein) concentrations were measured using a Qubit 4 Fluorometer (Thermo Fisher, Waltham, MA, USA). Selected concentrations of solubilized crystal protein solutions (75 µL) were applied to each well ensuring uniform distribution. Crystal solubilization buffer (50 mM Na_2_CO_3_) without 2-mercaptoethanol was used as control treatment. After drying, five neonates were introduced into each well before sealing with adhesive tray lids (CD International, Pitman, NJ). Trays were incubated at 27 °C, 21% relative humidity, and 16-h light/8-h dark photoperiod and mortality recorded after 7 days. Bioassays were replicated three times with 20 larvae tested per each concentration. Further bioassays were performed with larvae from susceptible and Dipel-resistant (NO-QAGE) strains of *P. xylostella* to estimate median lethal concentration (LC_50_) of solubilized crystal proteins from the most active *B. thuringiensis* strains in bioassays above. These bioassays were conducted as above, except for testing eleven crystal protein concentrations against 50 larvae from each strain per concentration, and bioassays replicated three times with larvae from different cohorts. Mortality data were analyzed by probit analysis using PoloPlus 1.0 (PoloPlus, 1987) to estimate the LC_50_s and 95% fiducial limits. The ratio between LC_50_ of AB1 against the susceptible and Dipel-resistant *P. xyslotella* strains and approximate 95% confidential boundaries were estimated as described elsewhere (Robertson et al., 2007).

### Genome sequencing and assembly

Genomic DNA of *B. thuringiensis* strain AB1 was purified as described above. DNA was quantified using Qubit 4 fluorometer (Thermo Fischer Scientific, Waltham, MA, USA) and randomly sheared using Covaris M220 Focused-ultrasonicator (Covaris, Woburn, MA, USA) to sizes between 200 and 500 bp per the manufacturer’s instructions (Barchenger et al., 2017). PCR free Illumina libraries were constructed using KAPA Hyper Prep Kit (Kapa Biosystems, Roche, Basel, Switzerland) followed by library quantification by qPCR KAPA Library Quantification Kit according to manufacturer’s instructions. The library was sequenced on Illumina Hiseq X at Admera Health (Illumina, San Diego, CA, USA). High throughput sequencing generated 38,737,140 high quality reads with an average length of 151 bp. The raw reads were deposited into Sequence Read Archive (SRA) database under NCBI accession PRJNA514414. Raw reads were quality trimmed and assembled (*de novo)* using CLC Genomic Workbench 11.0.1 software (Qiagen Bioinformatics, Redwood City, CA). *De novo* assembly of paired reads resulted in 213 contigs (N50 = 67 kbp). The assembled genome was submitted to the Prokaryotic GeneFinding tool in Blast2Go ver. 5.2.1 (Conesa et al., 2005) to obtain a FASTA file listing all possible Open Reading Frames (ORFs) in the contigs, which was used for proteomic analysis (see below). Homologous sequences to the predicted ORFs were searched using BLASTX in CloudBlast of Blast2Go software. GO terms were then assigned to each BLAST hit sequence by performing Mapping function followed by Annotation function, where the sequences were annotated using an annotation rule to the assigned GO’s using Blast2Go.

### Typing of *Bacillus thuringiensis* strain AB1

Multilocus sequence typing (MLST) analysis was performed using the PubMLST *Bacillus cereus* database (https://pubmlst.org/bcereus/) to identify the sequence type (genetically similar subspecies) of strain AB1. The AB1 genome was queried to identify the seven sequence type loci (*glpF*, *gmk*, *ilvD*, *pta*, *pur*, *pycA* and *tpi*) used for MLST with *B. thuringiensis* strains (Wang et al., 2018). The PubMLST database was then queried to determine the allele pattern for the seven loci, which matched the profile of sequence type 15 (ST15).

The full-length *GyrB* gene sequence from strain AB1 was identified as contig 122 in the AB1 genome using a BLAST in CLC Genomics Workbench ver. 11 (Qiagen Bioinformatics, Redwood City, CA, USA). Contig 122 was used as query in a BLAST search against NCBInr, and the 60 best matching coding sequences were used for sequence alignment in CLC Genomics Workbench. A neighbor joining phylogenetic tree was constructed using the aligned sequences. Stability of each node was assessed by bootstrap analysis with 1,000 replicates, and only bootstrap values >90% were considered for branches in the graphical representation of the tree.

### Proteomic identification of crystal proteins

Proteins in solubilized isolated parasporal crystal samples were identified and relatively quantified using SDS-10% PAGE and nano liquid chromatography followed by tandem mass spectrometry (nano LC/MS/MS). Solubilized crystal proteins (10 µg) were resolved by Bis-Tris SDS-10% PAGE, the gel stained with Coommassie blue, and the entire mobility region excised. The gel piece was then submitted to MS Bioworks LLC (Ann Arbor, MI) for nano LC/MS/MS. Trypsin digestion was performed by washing the gel piece with 25 mM ammonium bicarbonate followed by acetonitrile. Gel was reduced with 10 mM dithiothreitol at 60 °C followed by alkylation with 50 mM iodoacetamide at room temperature and digested with trypsin for 4 h at 37 °C. Trypsin activity was quenched with formic acid. The processed protein sample (supernatant) was analyzed by nano LC/MS/MS in a Waters NanoAcquity HPLC system interfaced to a LTQ Orbitrap Velos mass spectrometer (Thermo Fisher Scientific, Waltham, MA, USA). Peptides were loaded on a trapping column and eluted over a 75 µM analytical column at 350 nL/min; both columns were packed with Luna C18 resin (Phenomenex, Torrance, CA, USA). The mass spectrometer was operated in data-dependent mode, with MS and MS/MS performed in the Orbitrap at 70,000 FWHM resolution and 17,500 FWHM resolution, respectively. The fifteen most abundant ions were selected for MS/MS. Data were queried against a database including all the translated protein sequences from predicted open reading frames identified in the *B. thuringiensis * AB1 strain genome through the Prokaryotic GeneFinding tool in Blast2Go, as described above. Querying was performed using the Mascot software (Matrix Science, London) with carbamidomethyl (C) as fixed modification, and oxidation (M), acetyl (protein N-terminus), and deamidation (NQ) as variable modifications. Additional parameters included 10 ppm peptide mass tolerance, 0.02 Da fragment mass tolerance and two maximum missed cleavages. Mascot DAT files were parsed into the Scaffold ver. 4.8.8 (Proteome Software Inc., Portland, OR, USA) for validation based on peptide identification, filtering and to create a non-redundant list. Peptide identifications were accepted if they could be established at greater than 95.0% probability by the Scaffold Local FDR algorithm. Protein identifications were accepted if they could be established at greater than 99.0% probability and contained at least 2 identified peptides. Protein probabilities were assigned by the Protein Prophet algorithm (Nesvizhskii et al., 2003). Proteins that contained similar peptides and could not be differentiated based on MS/MS analysis alone were grouped to satisfy the principles of parsimony. The list of identified proteins was organized based on protein abundance considering the Normalized Spectral Abundance Factor (NASF) quantitative method (Zhang et al., 2010) which takes into account normalized spectra and protein length.

## Results

### Isolation and identification of *Bacillus thuringiensis* strains from Sri Lanka

**Figure 1:**
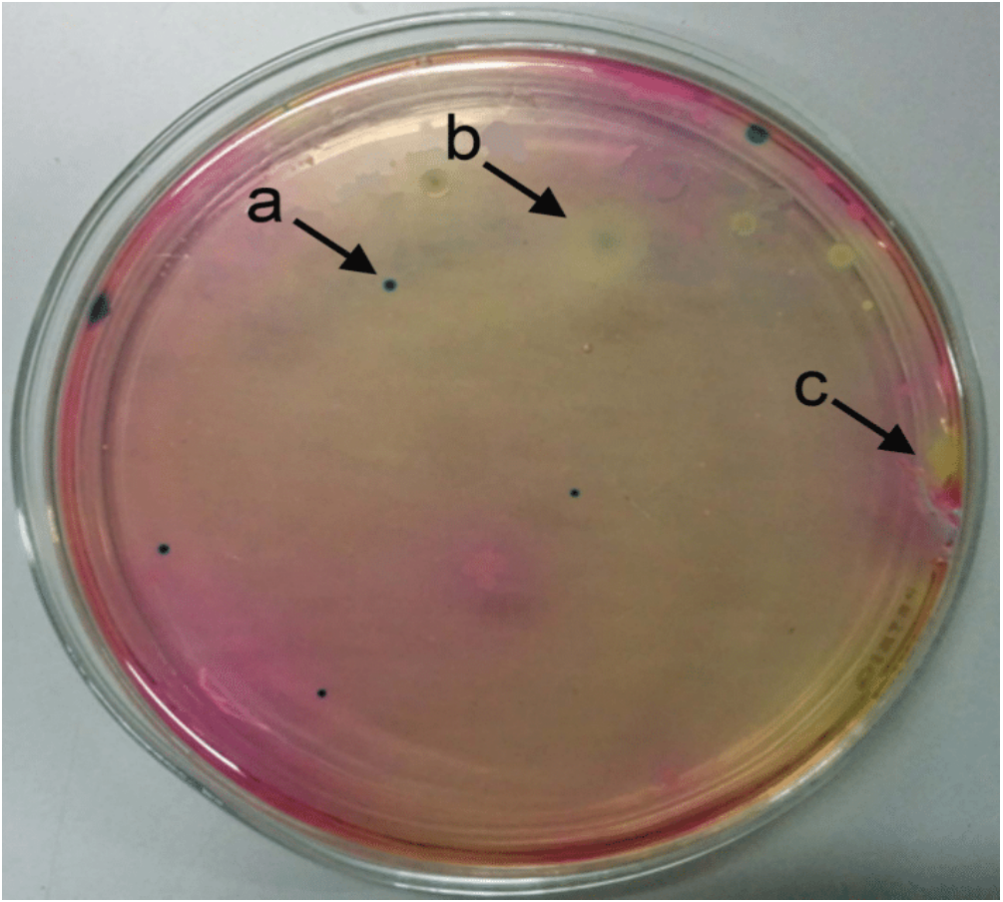
Selective identification of *Bacillus thuringiensis* colonies on *Bacillus* agar based on colony color. Selective identification of *Bacillus thuringiensis* colonies on *Bacillus* agar. *Bacillus thuringiensis* colonies were grown as blue/green in color (A), *Bacillus cereus* appeared as white color colonies with a light blue center (B), and *Bacillus megaterium* colonies appeared yellow (C).

Twenty-one *B. thuringiensis* strains were isolated from sixty-three soil samples collected from natural habitats in Sri Lanka (Table 1). For rapid and efficient isolation of Bt, we combined the conventional Travers method (Travers, Martin & Reichelderfer, 1987) with chromogenic identification of Bt as blue/green color colonies in *Bacillus* agar (HiChrome) (Fig. 1), making the identification of Bt from other spore-forming *Bacilli* much faster and easier.

**Table 1:** Insecticidal gene profiles of *Bacillus thuringiensis* strains isolated in Sri Lanka, with ICPs determined by visual detection and denoted by +.

***Bacillus thuringiensis*strain**	**GenBank*gyrB*gene accession number**	**Insecticidal gene profile**	**ICPs**
AB1	KF460446	*Cry* 1, *Cry4, Cry* 9, *Cry* 10, *Cry* 11, *Vip* 3A	+
AB2	KF481970.1	*Cry* 2, *Cry4, Cry* 9, *Cry* 10, *Cry11*	+
AB6	KX758551	*Cry* 1, *Cry* 9, *Cry* 10	–
AB7	KX758555	*Cry* 1, *Cry* 9, *Cry* 10, *Vip* 3A	–
AB8	KX758556	*Cry* 1, *Cry* 10, *Vip* 3A, *Cyt* 1	+
AB10	KX758557	*Cry* 1, *Cry* 9, *Cry* 10, *Cyt* 1	–
AB11	KX758553	*Cry* 9, *Cry* 10	–
AB12	KX758558	*Cry* 8, *Cry* 9, *Cry* 10	–
AB13	KX768535	*Cry* 9, *Cry* 10	–
AB14	KX768536	*Cry* 1, *Cry* 8, *Cry* 10, *Cyt* 1	–
AB15	KX758552	*Cry* 1, *Cry* 2, *Cry* 8, *Vip* 3A	+
AB16	KX758554	*Cry* 1, *Cry* 2, *Vip* 3A	–
AB17	KX758548	*Cry* 1, *Cry* 2, *Cry* 9, *Cry* 10, *Vip* 3A	–
AB19	KX758549	*Cry* 9, *Vip* 3A	–
AB20	KX758545	*Cry* 8	+
AB21	KX758547	*Cry* 9, *Cry* 10	+
AB22	KX758544	*Cry* 1, *Cry* 2, *Cry* 9, *Vip* 3A, *Cyt* 2	+
AB23	KX758546	*Cry* 9, *Cry* 10, *Cyt* 1	–
AB24	KX758550	*Cry* 9, *Cry* 10, *Cyt* 1	+
AB125	KF444206	*Cry* 2, *Cry* 4, *Cry* 9, *Cry* 10, *Cry* 11	+
AB142	KF444205	*Cry* 1, *Cry* 4, *Cry* 10, *Cry* 11, *Vip* 3A	+

Sequencing of the *gyrB* gene further identified the strains as Bt. Amplified *gyrB* sequences aligned with the *gyrB* gene sequences of reported *B. thuringiensis* strains available in the GenBank database with 99–100% sequence similarity. Sequence data have been deposited in GenBank (Table 1). The *gyrB* sequence data showed that the most common subspecies among the 21 tested Bt strains were *graciosiensis* (eight strains), *kurstaki* (five), *konkukian* (two) and *israelensis* (two).

Detection of insecticidal genes by PCR was used to predict insecticidal activity of native *B. thuringiensis* strains (Carozzi et al., 1991). Analysis of the 21 *B. thuringiensis* strains revealed the presence of amplified fragments characteristic of *cry*1, *cry*2, *cry*4, *cry*8*, cry*9, *cry*10, *cry*11, *vip*3A, *cyt*1 and *cyt*2 genes (Table 1). The most abundant (76% of strains) insecticidal gene among the screened *B. thuringiensis* strains was the dipteran-specific *cry*10. Among the lepidopteran-specific genes, *cry*1, *cry2* and *cry*9 genes were present in 52%, 28% and 71% of the tested strains, respectively, while *vip*3A gene was present in 42% of the strains. The frequency of dipteran-specific *cry*4/*cry*11, *cyt*1 and *cyt*2 genes was 19%, 23% and 4%, respectively. Nineteen percent of strains harbored the coleopteran-specific *cry*8 gene.

**Figure 2:**
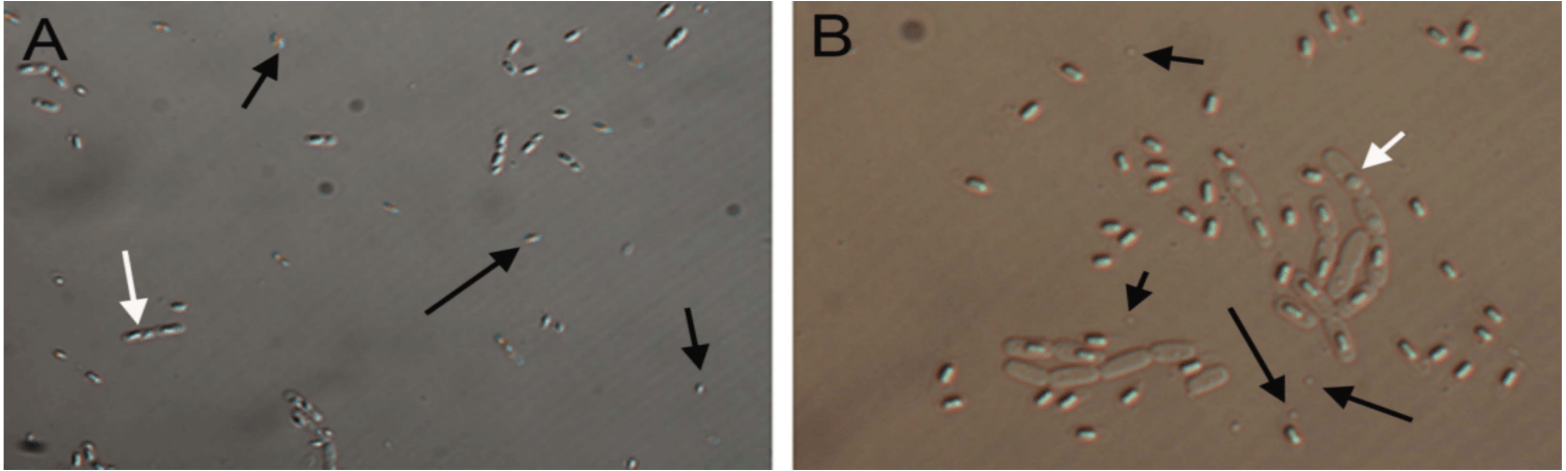
Differential Interference Contrast micrographs of *Bacillus thuringiensis* strains showing different morphologies of insecticidal crystal proteins (ICPs). Differential Interference Contrast micrographs of *Bacillus thuringiensis* strains showing rhomboidal parasporal crystals found in strain AB1 (A) and spherical crystals observed in strain AB21 (B). Black arrows point to released crystals, white arrows point to spores.

In contrast to detection of ICP genes in all tested strains, not all *B. thuringiensis* strains produced observable parasporal crystal proteins (Table 1). In fact, only 5 (AB1, AB2, AB8, AB125, AB142) out of the 21 strains showed high amounts of clear crystal inclusions. Lower levels of crystals inclusions were observed in strains AB21, AB22 and AB24, while crystals were not detected in the remaining strains. Different morphologies, including rhomboidal, spherical and spore-attached crystals were observed (Fig. 2).

### Toxicity of *Bacillus thuringiensis* strains against *Plutella xylostella*

**Figure 3:**
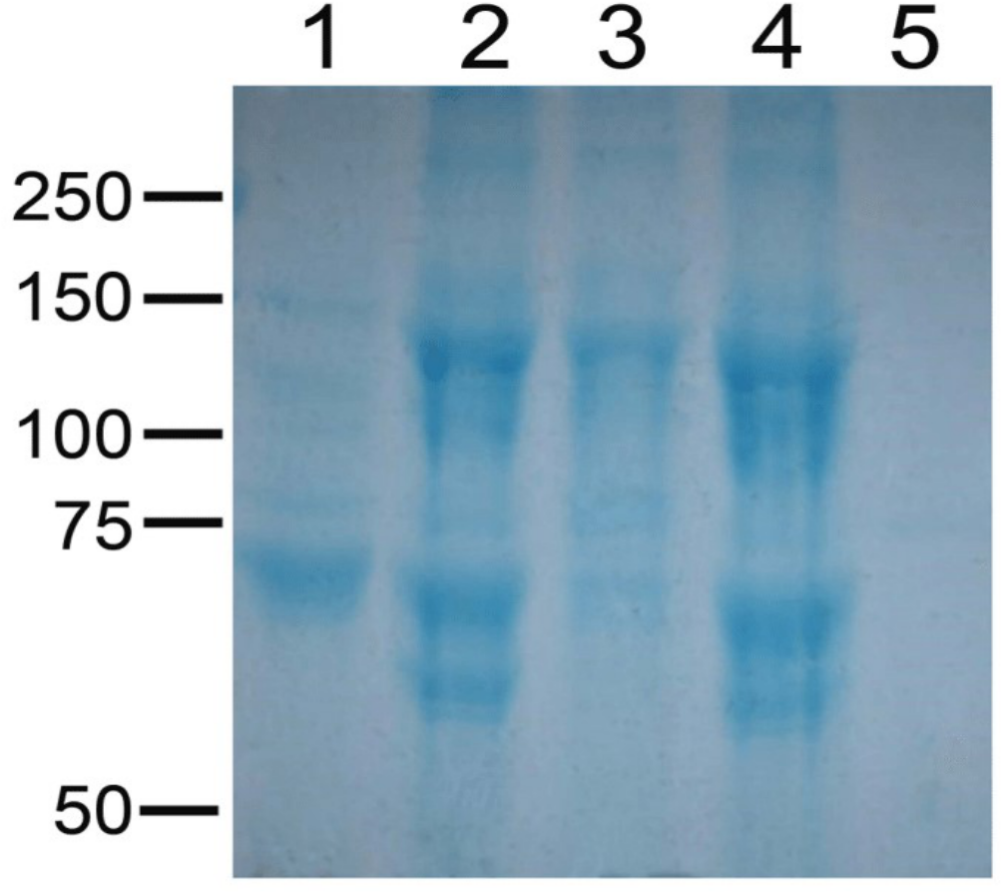
Electrophoretic pattern of solubilized crystal protein fractions from selected strains of *Bacillus thuringiensis*. Electrophoretic pattern of solubilized crystal protein fractions of *Bacillus thuringiensis* strain AB1 (lane 1), *Bacillus thuringiensis* strain AB2 (lane 2), *Bacillus thuringiensis* strain AB8 (lane 3), *Bacillus thuringiensis* strain AB125 (lane 4) and *Bacillus thuringiensis* strain AB142 (lane 5).

Due to limited resources, we focused toxicity assays on *B. thuringiensis* strains classified as producing high amounts of crystals (AB1, AB2, AB8, AB125 and AB142). The composition of the solubilized crystal protein fractions was assessed by electrophoresis (Fig. 3). Diversity in protein profiles were observed among the tested strains, in agreement with the diverse toxin genes detected by PCR in each strain.

**Figure 4:**
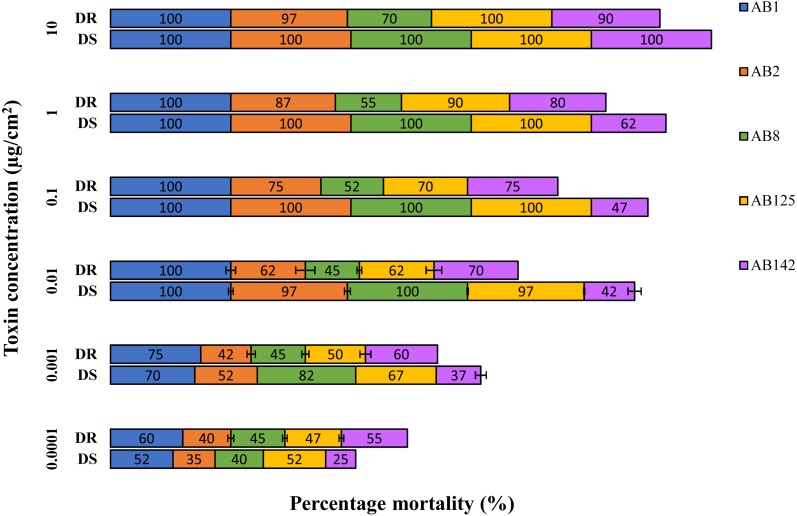
Toxic effect of isolated crystal proteins from *Bacillus thuringiensis* strains to neonates of Dipel-resistant-DR (NO-QAGE) and susceptible DS *Plutella xylostella* strains. Data represent mean mortality (%) ± SEM of 3 independent experiments.

Toxicity of the solubilized crystal protein fractions was assessed using a range of protein concentrations (from 0.1 µg to 10 µg /cm^2^) against larvae from Dipel susceptible and resistant populations of *P. xylostella* (Table 2). Percentage mortality data from this preliminary screen identified solubilized crystal proteins from *B. thuringiensis* strain AB1 as the most active against both susceptible and Dipel-resistant (NO-QAGE) strains of *P. xylostella*. Solubilized crystal proteins from strains AB2, AB8 and AB125 displayed similar toxicity against susceptible *P. xylostella* compared to AB1, but they did not present comparable toxicity against larvae from the NO-QAGE strain. *B. thuringiensis* strain AB142 displayed the lowest levels of activity against both strains of *P. xylostella* tested (Fig. 4).

**Table 2:** Toxicity parameters of *Bacillus thuringiensis* AB1 strain against susceptible and Dipel-resistant (NO-QAGE) populations of *Plutella xylostella* generated by probit analysis.

***Plutella xylostella*population**	**LC**_**50**_^a^**(FL 95%)**^b^	**Slope ± SE**^c^	***χ*2 (df)**^c^	**Intercept**
**Dipel-susceptible**	0.31 (0.18–0.57)	0.630 ± 0.034	105.59 (27)	0.318
**NO-QAGE**	0.09 (0.01–0.50)	0.399 ± 0.037	149.91 (38)	0.415

**Notes.**

a Lethal concentrations (LC_50_) ng/cm^2^.

b 95% lower and upper fiducial limits (FL 95%).

c Standard error of slope.

d Goodness of fit (df, degrees of freedom).

### Characterization of *Bacillus thuringiensis* strain AB1

The AB1 strain of *B. thuringiensis* was selected for further characterization based on its relatively higher toxicity against larvae from the susceptible and NO-QAGE strains of *P. xylostella*. This strain was deposited and is archived in the *B. thuringiensis* strain collection at the Industrial Technology Institute in Colombo (Sri Lanka), with accession number ITI-SL-AB1-2013. The genome of the strain was sequenced using Hiseq Illumina to identify insecticidal protein genes present and to generate a custom database for proteomic querying in the identification of insecticidal proteins among the solubilized crystal proteins.

Concentration-mortality assays with the solubilized crystal proteins from *B. thuringiensis* strain AB1 were performed to estimate toxicity parameters (Table 2). While not statistically significant due to overlapping fiducial limits values (Gonzalez-Cabrera et al., 2001; Mushtaq, Shakoori & Jurat-Fuentes, 2018), results from these bioassays identified a lower LC_50_ for these proteins against larvae from the NO-QAGE (0.091 µg/cm^2^) compared to the susceptible (0.313 µg/cm^2^) strain. The ratio of LC_50_ values and the corresponding 95% confidence intervals calculated as described by Robertson et al. (2007) suggested a 3.4-fold difference in susceptibility between the strains with 2.0 and 5.8 as lower and upper limits.

We used high throughput sequencing to further identify genes expressed in *B. thuringiensis* strain AB1. Assembly of reads resulted in 213 contigs with and N50 of 67 kb. Genome annotation results showed that *B. thuringiensis* strain AB1 contained pesticidal genes *cry1Aa*, *cry1Ca, cry1Da*, *cry1Ia*, *cry2Ab*, *cry9Ea* and *vip3A*.

Nano LC/MS/MS was performed on the solubilized crystal proteins of strain AB1 to identify the insecticidal genes expressed in the strain. Queries of MS data against the database of predicted ORFs from the genome confirmed the presence of 5 insecticidal Cry proteins in the AB1 sample (Table 3). Among these detected Cry toxins, Cry1Ca and Cry1Da were the most abundant, representing 61.2% and 31.2% of the total normalized spectra. The other three toxins detected, Cry1Aa, Cry1Ia, and Cry2Ab, represented <3% of the normalized spectra (Table 3).

**Table 3:** Insecticidal proteins in parasporal crystals of *Bacillus thuringiensis* strain AB1 as detected by nano LC/MS/MS against a database of predicted ORFs from the genome.

**Identified protein**	**Accession number**	**Description**	**EUPC**^a^	**NSAF**^b^
Cry1Ca	WP_042991625.1	Pesticidal protein	120	96.6
Cry1Da	WP_042991627.1	Pesticidal crystal protein Cry1Da	70	19.6
Cry1Aa	WP_00369823.1	Pesticidal crystal protein Cry1Aa	6	0.671
Cry1Ia	WP_000769223.1	Pesticidal crystal protein Cry1Ia	3	0.471
Cry2Ab	WP_001089638.1	Pesticidal crystal protein Cry2Ab	3	0.402

**Notes.**

a Exclusive Unique Peptide Count.

b Normalized Spectral Abundance Factor (×1,000), calculated using the number of spectra divided by the protein length and then normalized over the total of spectral counts/length for all the proteins in the sample.

### Typing of *Bacillus thuringiensis* strain AB1

Multilocus sequence typing using the AB1 genome in the PubMLST database identified the allele pattern for the seven loci used for classification: nine (*glp*), eight (gmk), 16 (*ilv*), 13 (*pta*), two (*pur*), 16 (*pyc* A) and nine (*tpi*), which matched the profile of sequence type 15 (ST15). This ST15 group comprises 12 bacterial isolates, of which eight are Bt. Seven out of the eight Bt isolates belong to serovar *aizawai*, supporting that strain AB1 belongs to this serovar.

**Figure 5:**
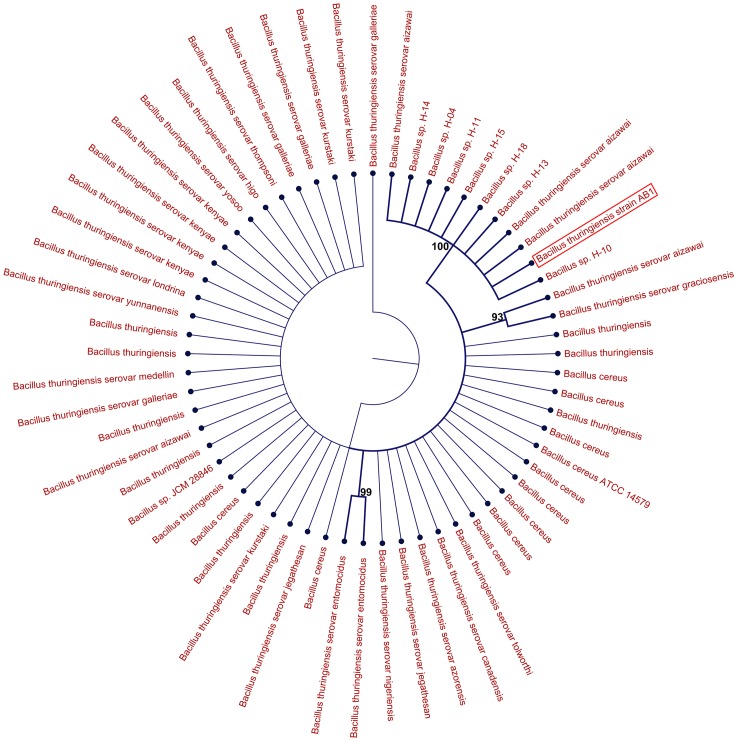
Neighbor-joining tree displaying identity in the *gyrB* gene sequence between the AB1 strain and the 60 most closely identical *gyrB* gene sequences in the NCBInr database. Stability of each node was assessed by bootstrap analysis with 1,000 replicates, and only bootstrap values >90% were considered for branches in the graphical representation of the tree.

Further evidence for serotyping of strain AB1 as *aizawai* was obtained through analysis of the full length *gyrB* gene sequence. A neighbor-joining tree constructed using the *gyrB* sequence from the strain AB1 genome (contig 122) and the 60 most similar *gyrB* sequences in the NCBInr database (Fig. 5), identified different clades (only considering branches with bootstrap values >90%). Strain AB1 was clustered in a clade together with strains of *Bacillus thuringiensis* serovar *aizawai*.

## Discussion

Biopesticides based on the bacterium Bt account for about 80% of the microbial pesticide market and represent a safer and more environmentally friendly approach alternative to synthetic pesticides, which display broad toxicity. These Bt pesticides have been used for decades for the control of *P. xylostella* larvae, the most devastating pest of crucifer crops worldwide (Sarfraz, Keddie & Dosdall, 2005). Intensive use of Bt pesticides to control *P. xylostella* has resulted in evolution of resistance in populations at diverse locations worldwide (Tabashnik et al., 1996; Heckel et al., 2001) highlighting the importance of discovering new *B. thuringiensis* strains that overcome resistance. In this regard, we report identification of *B. thuringiensis* strain AB1 isolated from Sri Lankan soil as toxic against susceptible and Dipel-resistant strains of *P. xylostella*.

Among 21 Bt strains characterized, strain AB1 displayed the highest relative levels of activity against *P. xylostella*. Interestingly, strains AB2 and AB8 displayed similar levels of activity against susceptible *P. xylostella* but were not as active against the NO-QAGE larvae. Based on these observations, we limited detailed characterization to strain AB1.

Both MLST and analysis of the full length *gyrB* gene typed strain AB1 as *B. thuringiensis* subsp. *aizawai*. In agreement with this conclusion, analysis of the flagellin gene showed highest similarity of AB1 to *B. thuringiensis* subsp. *aizawai* (ETE87447). In addition, the insecticidal gene composition of strain AB1 mostly resembles *B. thuringiensis* subsp. *aizawai* HD-133 (Jeong, Choi & Park, 2017). All these observations strongly support typing of strain AB1 as *B. thuringiensis* serovar *aizawai*.

Comparison of genomic and proteomic results support that the AB1 strain produces Cry1Aa, Cry1Ca, Cry1Da, Cry1Ia, and Cry2Ab proteins, albeit at diverse levels. The high level of toxicity of crystal proteins from strain AB1 against larvae from susceptible and NO-QAGE strains of *P. xylostella* is explained by the insecticidal crystal proteins produced by this strain. Proteomic data supported that Cry1C and Cry1D proteins make up to approximately 92% of the insecticidal crystal proteins in AB1. Neither of these toxins share binding sites with Cry1Ac in *P. xylostella* or any other lepidopteran larvae (Jakka, Ferré & Jurat-Fuentes, 2015), and they do not display cross-resistance in Cry1C- and Cry1D-resistant *P. xylostella* (Sayyed & Wright, 2001). Thus, “Mode 1” resistance in NO-QAGE, characterized for high resistance levels to at least one Cry1A protein and low or nil cross-resistance to Cry1C, is overcome by Cry1Ca and Cry1D in parasporal crystals from strain AB1. Attempts to sequence the *cry1C* gene in AB1 strain confirmed that it is >99% identical to the *cry1Ca* gene—JN651493.1 (data not shown).

Trace levels of Cry2Ab and Cry1I proteins were detected (three unique peptides matching to each) in the parasporal crystal proteome of strain AB1. Genes in both the Cry2Ab and Cry1I families are generally cryptic in nature (Tailor et al., 1992; Crickmore, Wheeler & Ellar, 1994), which probably explains the trace amounts detected for both proteins.

## Conclusions

Overall, findings of this study identify a native Sri Lankan strain of *B. thuringiensis* (AB1) typed as serovar *aizawai* producing mostly Cry1Ca and Cry1D toxins in its parasporal crystals. Data from this study support the potential use of this new *B. thuringiensis* subsp. *aizawai* strain AB1 to control *P. xylostella* larvae and to delay evolution of resistance to Bt pesticides that rely on Cry1A toxicity for their efficacy. Further work will be needed to determine the feasibility of culturing *B. thuringiensis* subsp. *aizawai* strain AB1 for commercialization.

##  Supplemental Information

10.7717/peerj.7535/supp-1 Table S1 Primers used for the screening of insecticidal genes

## Additional Information and Declarations

### DNA Deposition

The following information was supplied regarding the deposition of DNA sequences: Data is available at GenBank, accession number: PRJNA514414.

### Data Availability

The following information was supplied regarding data availability: The measurements for toxicity are available as Table 2 and Fig. 4. The raw data summarized in Fig. 4 shows the AB1 as the most effective tested strain against susceptible and Dipel-resistant strains of *Plutella xylostella*. The raw mortality data of AB1 strain was used for determination of the lethal concentration killing 50% of individuals (LC_50_) of susceptible and Dipel-resistant *Plutella xylostella* strains and the other toxicity parameters shown in Table 2.

## References

[ref-1] Abbott Laboratories (1992). B.t. Products Manual.

[ref-2] Barchenger D, Lamour K, Sheu Z, Shrestha S, Kumar S, Lin S, Burlakoti R, Bosland P (2017). Intra and intergenomic variation of ploidy and clonality characterize *Phytophthora capsici* on capsicum sp. in Taiwan. Mycological Progress.

[ref-3] Ben-Dov E, Wang Q, Zaritsky A, Manasherob R, Barak Z, Schneider B, Khamraev A, Baizhanov M, Glupov V, Margalith Y (1999). Multiplex PCR screening to detect cry9 genes in *Bacillus thuringiensis* strains. Applied and Environmental Microbiology.

[ref-4] Ben-Dov E, Zaritsky A, Dahan E, Barak Z, Sinai R, Manasherob R, Khamraev A, Troitskaya E, Dubitsky A, Berezina N, Margalith Y (1997). Extended screening by PCR for seven cry-group genes from field-collected strains of *Bacillus thuringiensis*. Applied and Environmental Microbiology.

[ref-5] Bravo A, Sarabia S, Lopez L, Ontiveros H, Abarca C, Ortiz A, Ortiz M, Lina L, Villalobos FJ, Peña G, Nuñez Valdez ME, Soberón M, Quintero R (1998). Characterization of cry genes in a Mexican *Bacillus thuringiensis* strain collection. Applied and Environmental Microbiology.

[ref-6] Carozzi NB, Kramer VC, Warren GW, Evola S, Koziel MG (1991). Prediction of insecticidal activity of *Bacillus thuringiensis* strains by polymerase chain reaction product profiles. Applied and Environmental Microbiology.

[ref-7] Conesa A, Götz S, García-Gómez JM, Terol J, Talón M, Robles M (2005). Blast2GO: a universal tool for annotation, visualization and analysis in functional genomics research. Bioinformatics.

[ref-8] Crickmore N, Wheeler VC, Ellar DJ (1994). Use of an operon fusion to induce expression and crystallisation of a *Bacillus thuringiensis* delta-endotoxin encoded by a cryptic gene. Molecular and General Genetics.

[ref-9] Ejiofor AO, Johnson T (2002). Physiological and molecular detection of crystalliferous *Bacillus thuringiensis* strains from habitats in the South Central United States. Journal of Industrial Microbiology and Biotechnology.

[ref-10] Ferre J, Real M, Rie J, Jansens S, Peferoen M (1991). Resistance to the *Bacillus thuringiensis* bioinsecticide in a field population of *Plutella xylostella* is due to a change in a midgut membrane receptor. Proceedings of the National Academy of Sciences of the United States of America.

[ref-11] Furlong MJ, Wright DJ, Dosdall LM (2013). Diamondback moth ecology and management: problems, progress, and prospects. Annual Review of Entomology.

[ref-12] Glare T, Caradus J, Gelernter W, Jackson T, Keyhani N, Köhl J, Marrone P, Morin L, Stewart A (2012). Have biopesticides come of age?. Trends in Biotechnology.

[ref-13] Gonzalez-Cabrera J, Herrero S, Sayyed AH, Escriche B, Liu YB, Meyer SK, Wright DJ, Tabashnik BE, Ferre J (2001). Variation in susceptibility to *Bacillus thuringiensis* toxins among unselected strains of *Plutella xylostella*. Applied and Environmental Microbiology.

[ref-14] Heckel D, Tabashnik B, Liu Y, Gahan L, Anthony M, Zhao J, Baxter S (2001). Diamondback moth resistance to Bt: relevance of genetics and molecular biology to detection and management.

[ref-15] Höfte H, Whiteley HR (1989). Insecticidal crystal proteins of *Bacillus thuringiensis*. Microbiology Reviews.

[ref-16] Jakka S, Ferré J, Jurat-Fuentes J, Soberón M, Gao Y, Bravo A (2015). Cry toxin binding site models and their use in strategies to delay resistance evolution. Bt resistance: characterization and strategies for GM crops producing *Bacillus thuringiensis* toxins.

[ref-17] Jeong H, Choi S-K, Park S-H (2017). Genome Sequences of *Bacillus thuringiensis* serovar kurstaki strain BP865 and *B. thuringiensis* serovar aizawai strain HD-133. American Society for Microbiology.

[ref-18] Jurat-Fuentes JL, Crickmore N (2017). Specificity determinants for Cry insecticidal proteins: insights from their mode of action. Journal of Invertebrate Pathology.

[ref-19] Kirsch K, Schmutterer H (1988). Low efficacy of a *Bacillus thuringiensis* (Berl.) formulation in controlling the diamondback moth, *Plutella xylostella* (L.), in the Philippines. Journal of Applied Entomology.

[ref-20] Machekano H, Mvumi BM, Nyamukondiwa C (2017). Diamondback moth, *Plutella xylostella* (L.) in Southern Africa: research trends, challenges and insights on sustainable management options. Sustainability.

[ref-21] Mushtaq R, Shakoori AR, Jurat-Fuentes JL (2018). Domain III of Cry1Ac is critical to binding and toxicity against soybean looper (*Chrysodeixis includens*) but not to velvetbean caterpillar (*Anticarsia gemmatalis*). Toxins.

[ref-22] Nesvizhskii A, Keller A, Kolker E, Aebersold R (2003). A statistical model for identifying proteins by tandem mass spectrometry. Analytical Chemistry.

[ref-23] Perera O, Willis J, Adang M, Jurat-fuentes J (2009). Cloning and characterization of the Cry1Ac-binding alkaline phosphatase (HvALP) from Heliothis virescens. Insect Biochemistry and Molecular Biology.

[ref-24] Porcar M, Juárez-Pérez V (2003). PCR-based identification of *Bacillus thuringiensis* pesticidal crystal genes. FEMS Microbiology Reviews.

[ref-25] Reyes-Ramırez A, Ibarra J (2008). Plasmid patterns of *Bacillus thuringiensis* type strains. Applied and Environmental Microbiology.

[ref-26] Robertson JL, Russell RM, Preisler HK, Savin NE (2007). Bioassays with arthropods.

[ref-27] Sarfraz M, Keddie AB, Dosdall LM (2005). Biological control of the diamondback moth, *Plutella xylostella*: a review. Biocontrol Science and Technology.

[ref-28] Sayyed A, Wright D (2001). Cross-resistance and inheritance of resistance to *Bacillus thuringiensis* toxin Cry1Ac in diamondback moth (*Plutella xylostella* L) from lowland Malaysia. Pest Management Science.

[ref-29] Soufiane B, Côté JC (2009). Discrimination among *Bacillus thuringiensis* H serotypes, serovars and strains based on 16S rRNA, gyrB and aroE gene sequence analyses. Antonie van Leeuwenhoek. International Journal of General and Molecular Microbiology.

[ref-30] Tabashnik BE, Cushing NL, Finson N, Johnson MW (1990). Field development of resistance to *Bacillus thuringiensis* in diamondback moth (Lepidoptera: Plutellidae). Journal of Enonomic Entomology.

[ref-31] Tabashnik B, Groeters F, Finson N, Liu Y, Johnson M, Heckel D, Luo K, Adang M, T Brown (1996). Resistance to *Bacillus thuringiensis* in *Plutella xylostella*: the moth heard round the world.

[ref-32] Tailor R, Tippett J, Gibb G, Pells S, Pike D, Jordan L, Ely S (1992). Identification and characterization of a novel *Bacillus thuringiensisδ*-endotoxin entomocidal to coleopteran and lepidopteran larvae. Molecular Microbiology.

[ref-33] Talekar NS, Shelton AM (1993). Biology, ecology and management of the Diamondback moth. Annual Reviews of Entomology.

[ref-34] Travers RS, Martin PAW, Reichelderfer CF (1987). Selective process for efficient isolation of soil *Bacillus* spp. Applied and Environmental Microbiology.

[ref-35] Wang K, Shu C, Soberon M, Zhang J (2018). Systematic characterization of *Bacillus* genetic stock center *Bacillus thuringiensis* strains using multi-locus sequence typing. Journal of Invertebrate Pathology.

[ref-36] Zalucki MP, Shabbir A, Silva R, Adamson D, Shu-Sheng L, Furlong MJ (2012). Estimating the economic cost of one of the world’s major insect pests, *Plutella xylostella* (Lepidoptera: Plutellidae): just how long is a piece of string?. Journal of Economic Entomology.

[ref-37] Zhang Y, Wen Z, Washburn MP, Florens L (2010). Refinements to label free proteome quantitation: how to deal with peptides shared by multiple proteins. Analytical Chemistry.

[ref-38] Zhao J, Cao J, Li Y, Collins H, Roush R, Earle E, Shelton A (2003). Transgenic plants expressing two *Bacillus thuringiensis* toxins delay insect resistance evolution. Nature Biotechnology.

[ref-39] Zhao J, Li Y, Collins H, Mau R, Thompson G, Shelton A (2002). Monitoring and characterization of Diamondback moth (*Lepidoptera: Plutellidae*) resistance to spinosad. Journal of Economic Entomology.

