# Supplementary material for: Identification of a native Bacillus thuringiensis strain from Sri Lanka active against Dipel-resistant Plutella xylostella
Source: PeerJ. 2019 Aug 23;7:e7535. doi: 10.7717/peerj.7535 (PMC6709662; doi:10.7717/peerj.7535)
Supplement: Table S1 [file peerj-07-7535-s001.docx]

**Supplementary Table 1:** Primers used for the screening of insecticidal genes

| **Primer**  **name** | **Primer sequence** | **Expected product size (bp)** | **Annealing**  **temperature**  **(^˚^C)** |
| --- | --- | --- | --- |
| *Cry*1F | 5' CATGATTCATGCGGCAGATAAAC 3' | 277 | 57 |
| *Cry*1R | 5' TTGTGACACTTCTGCTTCCCATT 3' |  |  |
| *Cry*2F | 5' GTTATTCTTAATGCAGATGAATGGG 3' | 700 | 55 |
| *Cry*2R | 5' CGGATAAAATAATCTGGGAAATAGT 3' |  |  |
| *Cry*3F | 5' CGTTATCGCAGAGAGATGACATTAAC 3' | 588 | 57 |
| *Cry*3R | 5' CATCTGTTGTTTCTGGAGGCAAT 3' |  |  |
| *Cry*4F | 5' CAAGCCGCAAATCTTGTGGA 3' | 797 | 58.5 |
| *Cry*4R | 5' ATGGCTTGTTTCGCTACATC 3' |  |  |
| *Cry*7 F | 5' GCGGAGTATTACAATAGAATCTATCC 3' | 916 | 40 |
| *Cry*7 R | 5' CTTCTAAACCTTGACTACTT 3' |  |  |
| *Cry*8 F | 5' ATGAGTCCAAATAATCTAAATG 3' | 376 | 57 |
| *Cry*8 R | 5' TTTGATTAATGAGTTCTTCCACTC 3' |  |  |
| *Cry*9F | 5' CGGTGTTACTATTAGCGAGGGCGG 3' | 350 | 52 |
| *Cry*9 R | 5' GTTTGAGCCGCTTCACAGCAATCC 3' |  |  |
| *Cry*10 F | 5' TCGTGGAATGGGCAAAAAC 3' | 404 | 58.5 |
| *Cry*10R | 5' TATCCCCCTTCAACATCCTCA 3' |  |  |
| *Cry*11 F | 5' TTTGCACCAGATAATACTAAGGAC 3' | 485 | 59.4 |
| *Cry*11 R | 5' AACAACTGCGATAAATACCACTCT 3' |  |  |
| *Vip*3A F | 5’ CCTCTATGTTGAGTGATGTA 3’ | 1029 | 56.8 |
| *Vip*3A R | 5’ CTATACTCCGCTTCACTTGA 3’ |  |  |
| *Cyt*1 F | 5’ CCGATGGGTGCTGTAGTGAG 3’ | 277 | 57.7 |
| *Cyt*1 R | 5’ CAGTTTGGGCATTTTGGATTG 3’ |  |  |
| *Cyt*2 F | 5’ ATCCGCCCATAATACAAG 3’ | 386 | 54.5 |
| *Cyt*2 R | 5’ GATACGGTTCACAGACG 3’ |  |  |
